# Comparison of the response of frequently relapsing steroid-dependent minimal change nephrotic syndrome to rituximab therapy between childhood-onset and adult-onset disease

**DOI:** 10.1097/MD.0000000000012704

**Published:** 2018-10-19

**Authors:** Yuko Iwabuchi, Yoei Miyabe, Shiho Makabe, Marie Nakano, Shun Manabe, Kazunori Karasawa, Takahito Moriyama, Kosaku Nitta

**Affiliations:** Department of Medicine, Kidney Center, Tokyo Women's Medical University, Tokyo, Japan.

**Keywords:** adverse effects, minimal change nephrotic syndrome, relapse, rituximab

## Abstract

Rituximab has been approved in Japan for the treatment of intractable nephrotic syndrome, but in cases of childhood-onset disease only; its efficacy and safety in adult-onset disease has yet to be established. This study was undertaken to evaluate the efficacy of rituximab and adverse effects in patients with adult-onset minimal change nephrotic syndrome (MCNS).

The study involved 32 childhood-onset cases (mean age at onset: 8.6 years) and 19 adult-onset cases (mean age at onset: 30.6 years) of frequently relapsing steroid-dependent MCNS, all of whom received intravenous rituximab drip infusion (375 mg/m^2^ body surface area per dose) at 4 time points at 6-month intervals. Relapse frequency, oral dose of immunosuppressants, and adverse effects were compared between the 2 groups.

Remission was maintained in all cases in the childhood-onset and adult-onset groups; a significant reduction in relapse frequency was noted during the first 24 months of rituximab therapy (0.3 ± 0.7 times and 0.3 ± 0.6 times in the childhood-onset and adult-onset groups, respectively; *P* < .001). Oral corticosteroid therapy could be discontinued in 81.3% of cases of the childhood-onset group (26/32 cases) and in 70.6% of cases of the adult-onset group (12/17 cases); the oral corticosteroid dose was reduced significantly to 0.9 ± 2.5 mg/day in the childhood-onset group and to 0.8 ± 1.6 mg/day in the adult-onset group (*P* < .001). Cyclosporin treatment was also discontinued in 87.5% of cases in the childhood-onset group (21/24 cases) and in 80.0% of cases of the adult-onset group (15/21 cases); the oral cyclosporin dose was reduced significantly to 8.6 ± 27.4 mg/day and 9.2 ± 22.0 mg/day, respectively (*P* < .001). Regarding adverse reactions, infusion reactions developed at a frequency of 21.1% and 19.7% in both groups, respectively, with no significant inter-group difference (*P* = .72).

Rituximab showed significant efficacy in adult-onset MCNS, with a comparable incidence of adverse reactions to that in childhood-onset cases, suggesting that this drug can also be used safely in adult-onset MCNS.

## 1 Introduction

Frequently relapsing steroid-dependent nephrotic syndrome often requires prolonged treatment with prednisolone (PRED) and inevitably necessitates administration of large doses of PRED. For this reason, adverse reactions to PRED pose a serious issue in the treatment of this disease. Among others, adverse reactions such as osteoporosis, femoral head necrosis, glaucoma, diabetes mellitus, and severe infections may not only reduce quality of life for patients, but also worsen the prognosis.

In the latter half of the 2000s, the efficacy of rituximab in reducing urinary protein excretion in patients with nephrotic syndrome was reported in Japan and overseas. Notable is a report in 2008 by Yang et al, which stated that adult-onset cases of minimal change nephrotic syndrome (MCNS) also responded to this therapy to a degree comparable to that of childhood-onset.^[1]^ We also previously demonstrated the efficacy of this drug in adult-onset MCNS.^[2,3]^ On the basis of previous reports, we first evaluated the efficacy of single-dose administration of rituximab (375 mg/kg/m^2^) repeated at 6-monthly intervals and the adverse reactions associated with this treatment in 24 patients with primary glomerulonephritis, including cases of frequently relapsing nephrotic syndrome. That evaluation revealed marked efficacy of the treatment in cases of MCNS, particularly in frequently relapsing cases of nephrotic syndrome, without any significant adverse effects. The findings additionally suggested that the disease is more likely to relapse in cases with elevated CD19 and 20 counts.^[4]^ Subsequently, we investigated the efficacy/safety of rituximab therapy for MCNS with the approval of the ethics committee of our facility in 2008, and demonstrated that this therapy allowed reduction of the dose/discontinuation of steroids, as well as discontinuation of the immunosuppressant agent used, accompanied by improvement in bone mineral density, without any serious adverse effects.^[5–7]^ In an analysis of the healthcare expenditure, rituximab therapy was revealed to be superior to existing treatment methods from the viewpoint of medical economy.^[8]^ Thus, rituximab was expected to have the following advantages when used for the treatment of steroid-dependent MCNS: lower incidence of relapse, reduction in the frequency of adverse reactions through its potential for steroid dose reduction/discontinuation, while facilitating prevention of complications, and economic advantage through the possibility of discontinuation of immunosuppressive agents used. In August 2014, the use of this drug in patients with intractable nephrotic syndrome (frequently relapsing or steroid-dependent type) was approved for coverage by national health insurance in Japan. However, this approval was limited to childhood-onset disease only, and the efficacy and safety of this drug in adult-onset disease remain to be clearly established.

Under these circumstances, the present study was undertaken to retrospectively evaluate the efficacy of rituximab and the adverse reactions associated with its use in MCNS patients at our department, divided into a childhood-onset group and an adult-onset group.

## 2 Methods

### 2.1 Study population

Patients who fulfilled the following criteria were enrolled in this study. Those diagnosed with steroid-dependent nephrotic syndrome were defined as the occurrence of relapse during tapering of the PRED dose or within 2 weeks of PRED discontinuation. Nephrotic syndrome was defined by a urinary protein excretion level of >3.5 g/day, serum albumin level of <3.0 g/dL, edema, and hyperlipidemia. Relapse was defined as recurrence of massive proteinuria (daily urinary protein excretion level of 3.5 g/day or a 3+ or 4+ result on the urinary dipstick test for albumin). Urinary protein was evaluated in terms of either 24-hour urinary protein or the urinary protein/creatinine ratio. The study involved 51 patients who had participated in our previous uncontrolled study of rituximab therapy for frequently relapsing steroid-dependent MCNS conducted between March 2008 and March 2016 at the Department of Nephrology, Tokyo Women's Medical University (UMIN000005231). When childhood onset was defined as disease onset at less than 18 years of age and adult onset was defined as disease onset at ≥18 years of age, there were 32 childhood-onset cases (28 males and 4 females) and 19 adult-onset cases (12 males and 7 females) in our study population.

### 2.2 Study design

The study was conducted with the approval of the Research Ethics Board of Tokyo Women's Medical University. All the patients gave written informed consent to participate in the study.

The treatment protocol was described previously.^[6]^ The first rituximab dose was administered via intravenous infusion at a single dose of 375 mg/m^2^. In order to minimize the occurrence of infusion reactions, we administered 4 mg of betamethasone, 20 mg of monoammonium glycyrrhizinate, and 200 mg of acetaminophen to the patients 30 minutes prior to rituximab infusion. The rituximab infusion was repeated 3 times at 6-month intervals, that is, at 6, 12, and 18 months after the first infusion.

The following laboratory parameters were examined in all patients. An attempt was made to taper the steroid dose/discontinue the steroid by 24 months after the first rituximab infusion, although no precise protocol was set for tapering the steroid dose. There was no restrictive cause in the trial protocol for discontinuation of either the immunosuppressants or the steroid. Patients were followed up for 24 months after the first rituximab infusion. B-cell depletion and repletion were defined as a peripheral blood CD19 count of <10 and >10/mm^3^, respectively.

### 2.3 Demographic and laboratory measurement

Height (cm), body weight (kg), body mass index (kg/m^2^), blood pressure (mm Hg), urine protein (g/day), serum albumin (g/dL), serum creatinine (mg/dL), estimated glomerular filtration rate (eGFR, mL/min/1.73 m^2^), low-density lipoprotein cholesterol (LDL-C, mg/dL), high-density lipoprotein cholesterol (mg/dL), triglyceride (mg/dL), immunoglobulin G (IgG, mg/dL), immunoglobulin A (mg/dL), immunoglobulin M (mg/dL), hemoglobin A1c (HbA1c, %) levels, white blood cell (/mm^3^) count, lymphocyte (/mm^3^) count, peripheral blood CD19 (/mm^3^) count, peripheral blood CD20 (/mm^3^) count, peripheral blood CD4/8, and bone density (T score at the L1–L4 levels of the lumbar spine measured by dual-energy X-ray absorptiometry) were evaluated at baseline and at the end of the 24-month observation period. Primary endpoints were the number of relapses and number of patients requiring PRED and/or immunosuppressants. Secondary endpoints were the adverse effects of rituximab.

### 2.4 Statistical analysis

Data were expressed as mean ± standard deviation or median. All analyzed variables were tested for distribution. The *t* test and analysis of variance were used for samples with a normal distribution and the Wilcoxon signed-rank test for samples with a skewed distribution, to analyze the differences in the laboratory data recorded between baseline and at 24 months after the first rituximab infusion. Categorical data were analyzed by the χ^2^ test. All statistical analyses were performed using the JMP 11 software (SAS Institute, Cary, NC). Statistical significance was set at *P* < .05. All analyses were performed at an independent biostatistics and data center (STATZ Institute, Inc., Tokyo, Japan).

## 3 Results

### 3.1 Patient population

Regarding the patient characteristics at study baseline, mean age at disease onset was 8.6 years in the childhood-onset group and 30.6 years in the adult-onset group (*P* < .001). Age at the start of rituximab therapy was significantly higher in the adult-onset group (36.0 years) than that in the childhood-onset group (25.3 years) (*P* = .001). The interval from disease onset to the start of rituximab therapy was significantly longer in the childhood-onset group (16.8 years) than in the adult-onset group (5.5 years) (*P* < .001). The frequency of disease relapse did not differ between the childhood-onset group (4.2 times) and the adult-onset group (4.3 times) (*P* = .88). The percentage of patients who received oral corticosteroid treatment was 100% in the childhood-onset group and 89.5% in the adult-onset group, while the oral steroid dose level did not differ between the 2 groups (27.0 mg/day in the childhood-onset group and 23.2 mg/day in the adult-onset group; *P* = .38).

In terms of the use of cyclosporine A (CyA), 24 of the 32 patients (75.0%) in the childhood-onset group and 15 of the 19 patients (78.9%) in the adult-onset group received oral CyA, although the dose of oral CyA did not differ significantly between the 2 groups (98.8 and 84.2 mg/day, respectively; *P* = .46). The percentage of patients who received oral mizoribine was significantly higher in the childhood-onset group (15/32, 46.9%) than that in the adult-onset group (3/19, 15.8%) (*P* = .04). There were scant differences between the 2 groups in terms of height, body weight, or serological parameters. Serum creatinine level was significantly higher in the adult-onset group (*P* = .038), while eGFR was significantly higher in the childhood-onset group (*P* < .001). Serum immunoglobulin A level was significantly lower in the childhood-onset group (*P* = .002).

### 3.2 Rituximab treatment

The frequency of disease relapse during the 24-month period after the start of rituximab treatment was 0.3 times in both the childhood-onset and adult-onset groups, this frequency being significantly lower than that recorded during the 24-month period before the start of rituximab treatment (*P* < .001). Oral corticosteroid treatment was successfully discontinued in 81.3% of all cases of the childhood-onset group and 70.6% of all cases of the adult-onset group, with a significant reduction of the mean oral corticosteroid dose level to 0.9 and 0.8 mg/day in the 2 groups, respectively (*P* < .001). In addition, CyA treatment was successfully discontinued in 87.5% of all cases of the childhood-onset group and 80.0% of all cases of the adult-onset group, with significant reduction of the mean oral CyA dose level to 8.6 and 9.2 mg/day in the 2 groups, respectively (*P* < .001). Furthermore, both groups showed significant elevation of serum albumin (*P* < .001) and IgG levels (*P* = .003, *P* = .001), and significant reduction of urinary protein excretion level (*P* < .001, *P* = .002), serum LDL-C level (*P* = .011, *P* = .024), and white blood cell count (*P* < .001). Furthermore, the blood count of B cells (CD19 cells) decreased significantly to 4.65/mm^3^ in the childhood-onset group and to 9.94/mm^3^ in the adult-onset group (*P* < .001), with no significant inter-group difference. In addition, significant improvement of T-score (*P* = .008) and significant reduction of serum levels of high-density lipoprotein cholesterol (*P* = .023) and immunoglobulin M (*P* < .001) were observed in the childhood-onset group. The adult-onset group showed significant improvement of blood pressure (systolic blood pressure, diastolic blood pressure; *P* = .03, *P* = .03) and HbA1c levels (*P* = .02).

### 3.3 Adverse events

In the childhood-onset group, infusion reactions developed in 17 patients after 27 of the 160 doses in total (21.1%). Infusion reactions are an adverse reaction that often appears within 24 hours after an infusion, and is characterized by symptoms such as pyrexia, chills, nausea, headache, pain, pruritus, and rash. In the adult-onset group, infusion reactions developed in 10 patients after 15 of the 76 doses in total (19.7%). Thus, the incidence of infusion reactions did not differ significantly between the 2 groups. Another adverse reaction, neutropenia, developed in 1 case each in the 2 groups.

## 4 Discussion

In the present study, rituximab therapy was associated with a reduced frequency of disease relapse and allowed dose reduction/discontinuation of steroids and immunosuppressants in both the childhood-onset group and adult-onset group. Ravani et al conducted a randomized controlled trial comparing rituximab + low-dose standard therapy (steroid + calcineurin inhibitor) with standard therapy in children with steroid- and calcineurin inhibitor-dependent primary nephrotic syndrome, and reported that the urinary protein excretion level decreased by 70% after 3 months of treatment in the rituximab + low-dose standard therapy group as compared to the standard therapy group.^[9]^ In Japan, Iijima et al conducted a double-blind randomized controlled trial in steroid-resistant cases and steroid-dependent cases, and reported that the frequency of relapse was significantly suppressed in the rituximab treatment group as compared to the placebo group.^[10]^ With regard to the efficacy in adult cases, in 2008, Yang et al published a report describing that the response of patients with adult-onset MCNS to the treatment was comparable in degree to that in childhood-onset cases.^[1]^ Munyentwali et al (2013), Ruggenenti et al (2014), and King et al (2017) reported the efficacy and safety of rituximab in adults with minimal change nephropathy, but their subjects included some childhood-onset cases as well. Thus, the present study is the first involving a group consisting entirely of adult-onset cases.^[11–13]^ In the present study, where there were fewer adult-onset patients (n = 19) than childhood-onset patients (n = 32), the frequency of relapse improved significantly (0.3 ± 0.6 relapses) following rituximab therapy in the adult-onset patients and was not inferior to that in the childhood-onset patients (*P* < .001). With regard to the doses of the oral medications, PRED and CyA were decreased to 0.8 ± 1.6 and 9.2 ± 22.0 mg/day, respectively, in the adult-onset patients, with no significant difference with the corresponding reductions in the childhood-onset patients (*P* < .001). Our results suggest sufficient efficacy of rituximab therapy in adult-onset patients as well.

PRED has serious adverse effects, such as obesity, growth retardation, hypertension, diabetes mellitus, and osteoporosis, and it has been reported that rituximab therapy allows the PRED dose, and also the severity of the aforementioned adverse effects, to be reduced. From a study of pediatric cases, Ruggenti et al reported that rituximab therapy resulted in the improvement of growth (height and body weight), blood pressure, and eGFR.^[12]^ We also demonstrated a significant improvement in the blood pressure, total cholesterol, HbA1c, IgG, CD4/8 ratio, and bone mineral density, and a significant reduction in the required doses of antihypertensive agents, drugs for the treatment of osteoporosis, and insulin following rituximab therapy in patients with MCNS.^[5–7]^ In the present study, significant elevation of serum IgG, a decrease in serum LDL-C, and improvement of leukocyte parameters were observed in both the childhood- and adult-onset patients and were thought to be attributable to amelioration of the immune responses and lipid metabolism consequent on the reduction of the PRED dose. Furthermore, a significant improvement of bone mineral density was evident in the childhood-onset patients and an improvement in blood pressure and impaired glucose tolerance was seen in the adult-onset patients, suggesting that rituximab therapy improved bone-mineral metabolism, water-electrolyte balance, and carbohydrate metabolism. Thus, rituximab therapy reduced the severity of complications in the adult-onset patients to an extent not inferior to that seen in the childhood-onset patients; therefore, sufficient efficacy may be expected from rituximab therapy in adult-onset patients as well Tables 1–3.

**Table 1:**
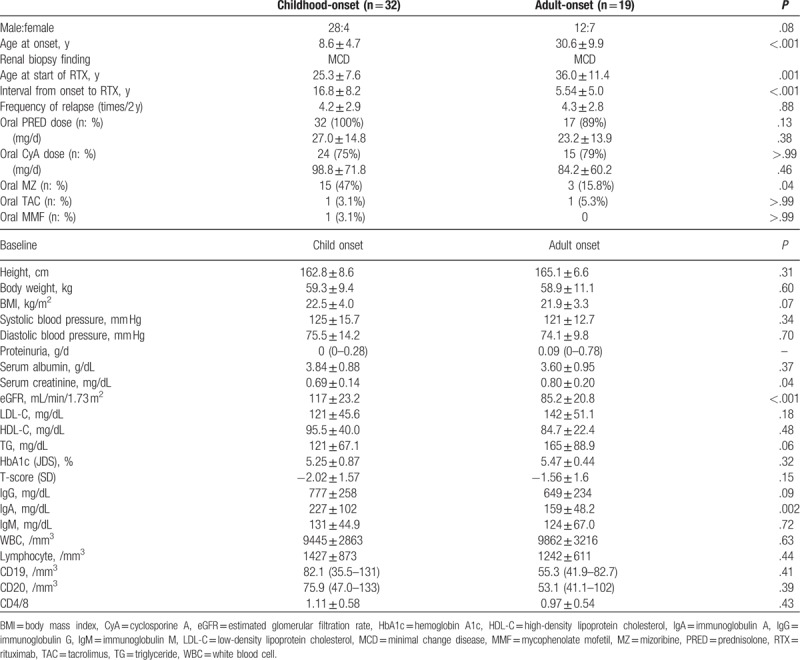
Baseline characteristics of study population.

**Table 2:**
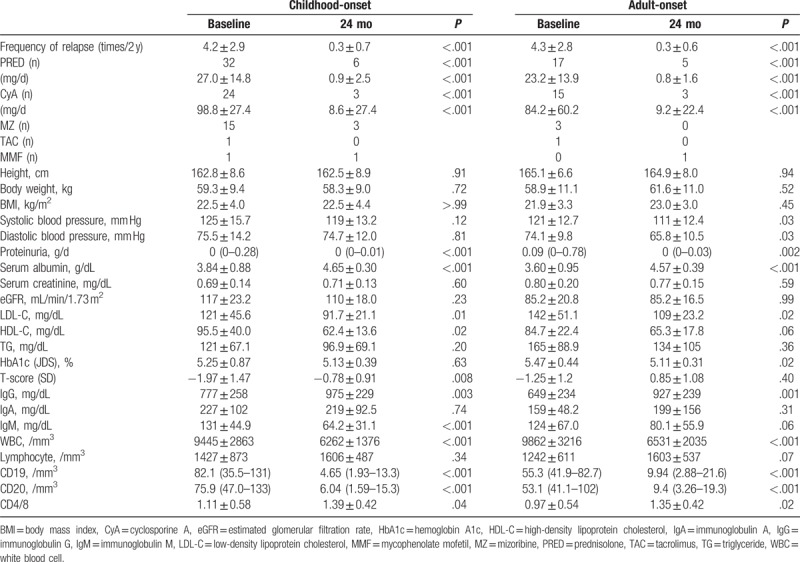
Comparison of parameters between baseline and after 24 mo of treatment.

**Table 3:**
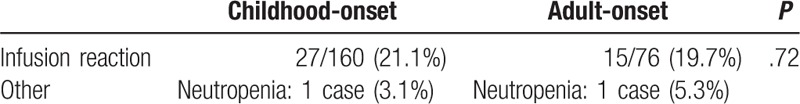
Adverse events.

Infusion reaction was the most frequently observed adverse reaction in this study. In a domestic phase III clinical trial of this drug conducted in patients with intractable nephrotic syndrome, infusion reaction was seen in 34 of the 54 subjects (63%) and was rated as grade II or less severe in all cases. The major manifestations of infusion reaction were hypertension (9 cases), dyspnea (8 cases), and pharyngeal discomfort (6 cases). This adverse reaction was more likely to occur during the first infusion, particularly during the 30- to 60-minute period after the first elevation of the infusion rate. In the present study, on the other hand, this reaction developed less frequently (21.1% in the childhood-onset group and 19.7% in the adult-onset group), and was rated as grade II or less severe in all cases. One possible reason for the lower incidence of this reaction in the present study could be the difference in the premedication protocol. The package insert for this drug includes a statement that primarily antihistamines and antipyretic analgesics should be used as premedication, and that addition of adrenocortical hormone preparations should be considered in only cases where such preparations are not being administered orally. In this study, all patients received premedication, including betamethasone 4 mg, acetaminophen 200 mg, and glycine/cysteine combination 20 mg for injection. Because betamethasone is known to have a longer half-life than methylprednisolone, we think the use of this corticosteroid with a longer half-life suppressed the development of infusion reaction within 24 hours after infusion. Regarding the occurrence of neutropenia, the clinical study mentioned above reported development of grade III or more severe neutropenia (neutrophils <1000/μL) at an incidence of 11.1%, while the incidence of this adverse reaction was much lower in the present study, that is, 1 case each in the childhood-onset group (3.1%) and adult-onset group (5.3%). In both cases, the reduced neutrophil count subsequently rose rapidly without necessitating administration of granulocyte-colony stimulating factor. Thus, the incidence of adverse reactions to rituximab did not differ between the childhood-onset group and the adult-onset group, suggesting that this drug can be used safely in both childhood-onset and adult-onset cases of this disease.

A limitation of this study was that it was not designed as a randomized study for comparison. It was difficult to enroll enough patients with frequently relapsing MCNS in a randomized comparative trial, which requires a large number of cases. Since the present study did not incorporate long-term follow-up after completion of rituximab therapy in the protocol, it was impracticable to make any comparative assessment of the long-term efficacy and adverse reactions between the childhood-onset patients and adult-onset patients. Nonetheless, the study demonstrated that rituximab therapy is effective in both childhood-onset and adult-onset MCNS, with no inter-group differences in the incidence and nature of adverse reactions.

In conclusion, rituximab therapy was shown to be significantly effective also in adult-onset nephrotic syndrome, with no increase in the frequency of adverse reactions as compared to that in childhood-onset, suggesting that this drug can also be used as safely in adult-onset as in childhood-onset disease.

## Acknowledgments

The authors thank the physicians who participated in this study namely Ari Shimizu, Masayo Sato, Toshio Mochizuki, Keiko Uchida, and Ken Tsuchiya (Tokyo Women's Medical University).

## Author contributions

**Conceptualization:** Yuko Iwabuchi, Kosaku Nitta.

**Data curation:** Yuko Iwabuchi, Yoei Miyabe, Shiho Makabe, Marie Nakano, Shun Manabe, Kazunori Karasawa, Takahito Moriyama.

**Investigation:** Yoei Miyabe, Shiho Makabe, Marie Nakano, Shun Manabe, Kazunori Karasawa, Takahito Moriyama.

**Methodology:** Kazunori Karasawa, Takahito Moriyama.

**Validation:** Yuko Iwabuchi, Takahito Moriyama.

**Writing – original draft:** Yuko Iwabuchi.

**Writing – review & editing:** Kosaku Nitta.
